# Liberating parents from guilt: a grounded theory study of parents’ internet communities for the recognition of ADHD

**DOI:** 10.1080/17482631.2018.1564520

**Published:** 2019-01-29

**Authors:** Nicolas Dauman, Marion Haza, Soly Erlandsson

**Affiliations:** a Department of Psychology, University of Poitiers, Poitiers, France; b Centre for Child and Youth Studies, University West, Trollhättan, Sweden

**Keywords:** Attention Deficit Hyperactivity Disorder (ADHD), parenting practices, guilty feelings, digital communities, internet, grounded theory, qualitative research

## Abstract

**Purpose**: This study presents a qualitative analysis of information posted on the Internet by two communities of French parents promoting the recognition of ADHD in the context of current health and school practices.

**Method**: Grounded Theory (Strauss & Corbin’s approach) was applied to the posted messages, with the aim to discover the main concern and common theme through a constant comparison analysis.

**Results**: Liberating parents from feeling responsible for their child’s misconduct was found to be the core category. From this perspective, we account for the commitment of the digital communities to formalize the child’s conduct as a consequence of a neurodevelopmental disorder. This approach helps to account for the promotion of behavioural expertise and conditioning strategies (e.g., positive reinforcement) for handling the child’s so-called disorder as appropriate parental responses. Giving evidence for parenting struggles was the third main concern of the communities, in the face of perceived skepticism from professionals towards ADHD as a medical condition.

**Conclusions**: By using examples from countries that are found to have a more pro-medical approach to ADHD, the communities aim at improving such medical practices in France. Issues surrounding the claim that ADHD would require a specific style of parenting are also discussed.

## Introduction

Parents of children with ADHD play a central role in the contemporary approach to understanding and managing their children’s behavioral difficulties. Moreover, according to Barkley (2013), parents’ associations for ADHD acknowledgement have contributed to the social recognition of this so-called disorder, whose prevalence was underestimated until the associations were initiated. Today, hundreds of thousands of parents who are greatly concerned about their child’s difficulties at school and in the family, are influenced by the recommendations of Internet forums defining a set of behaviors presumed to be signs of a “neurodevelopmental disorder.” With no consideration for these children’s emotional life or their social circumstances, parents are encouraged to demand an ADHD diagnosis and to undertake specific training to learn how to handle the child (Erlandsson et al., 2016; Meerman et al., 2017). Recently, parenting also became a focus of interest in studies suggesting that interactions between parents and children with ADHD are likely to contribute to psychosocial difficulties encountered in the family. Difficulties identified in the parenting of those who strive to manage their child’s ADHD can be linked to problems such as: the parent’s loss of confidence (Beaulieu & Normandeau, 2012; McLaughlin & Harrison, 2006), the rejection of their child (Oh et al., 2012) and a lack of interest in academics (Rogers et al., 2009). Thus, those parents would seem to be less able to contribute to improving their child’s health. In line with this perspective, parents of children with ADHD need to be taught efficient management strategies. Over the years, the disorder has become an issue that not only concerns the individual child but also the entire structure of the family, where relational difficulties must be addressed. Many programs offering management strategies and skills to parents also associate those skills with the managing of ADHD (e.g., Azevedo et al., 2014; Taylor et al. (2015); Stattin, 2015). For instance, Azevedo et al. (2014) used video recordings and role-playing to promote awareness of parenting practices among mothers who encountered difficulties in raising restless and demanding children. Aimed at helping parents to manage relational difficulties, such studies do not, however, question these difficulties as a cultural construct (Timimi & Taylor, 2004).

A contextualized understanding of ADHD should consider the role played by information and notions relating to health and the children’s misbehavior as well as the origin of this behavior. The access to Internet forums has deeply transformed the relationships between health professionals, researchers and patients as the latter now have access to medical data prior to their encounter with health institutions (Murray et al., 2003). Help-seeking individuals have become more active in their search for medical assistance, sometimes challenging clinicians’ knowledge of their condition. A major change resulting from the use of the Internet in the medical field is that patient’s expectations for clinical management of their health problems are becoming higher (McMullan, 2006). The digital revolution includes two significant social phenomena: increased access to information about medical diagnoses (Tang & Hwee Kwoon, 2006), and the emergence of digital, mutual-help communities related to the latter (Casilli, 2010; Scharer, 2005b). The practice of parenthood has been largely influenced by both these social phenomena. A search on Google using the keywords “parent and health” shows more than 90 million hits, with 50 million hits for the words: “parent, health, and children” (Daneback & Plantin, 2008). A wealth of information about particular diagnoses and treatments for childhood illnesses is available for any of the problems encountered by parents (Bernhardt & Felter, 2004; Oprescu et al., 2013). As noted by Plantin and Daneback (2009), today parents are looking for concrete information, enabling them to compare their experience to that of others. The search for information, based on experience, is strengthened in the parents of children suffering from mental illness, neurological disorders, or autism. They feel that social support provided over the Internet is particularly important (Leonard et al., 2004; Fleischmann, 2005; Scharer, 2005a). In addition to information offered by the physician, parents search the Internet for a second opinion, confirming their physician’s judgement (O’Connor & Madge, 2004) or to check if their child’s conduct is normal (Blackburn, 2005).

Studies focusing on digital communities have emphasized the significant difference between digital and traditional offline communities. The advantages of digital forums seem to be that they can influence the social environment through the pooling of individual actions (Smith & Kollock, 1999). Successful digital forums may also enable a faster return on the individual’s investment, that is, fast feedback from other members of the forum after spending time sharing experience and information (Casilli, 2010). A wider reciprocity and demonstration of support seems to be more rewarding than what can be offered by members of offline communities with prior, local experience. Furthermore, the Internet as a medium gives a sense of legitimacy to information that circulates in the communities (Cauquelin, 1999). It appears to be indisputable, especially when the user is not able to contextualize the information provided or distance her/himself from it (Dubey, 2001; Tisseron, 2008). Digital communities are likely to be a significant resource for people seeking advice and help for disorders that lack medical legitimacy. Hence, these communities are a source of research data aimed at understanding collective actions that are meant to aid in the acknowledgement of difficulties related to health issues. According to Lundin and Erlandsson (2017), considering the popularity of the support groups on the Internet, more research is needed to explore and comprehend the social processes that take place in these forums.

Although researchers from various disciplines have shown that it is inadequate to view ADHD as a neurobiological disorder the current medical discourse on ADHD reflects some kind of social consensus in many countries around the world. For example, it was suggested by Pedersen (2015) that the developing emphasis on biomedical markers and medication mimics that in the USA. Smith (2018) analysed, by the use of medical literature, how countries as Canada, the UK, Scandinavia, India and China have adopted ADHD as a neurobiological disorder and how the reception of ADHD in those countries varies. Although ADHD is a universal disorder and has been aggressively promoted by psychiatrists and pharmaceutical companies the picture varies; some countries embrace the ADHD disorder but there are also countries that have rejected the diagnosis (Smith, 2018). In general terms, surprisingly little criticism has been directed towards the biomedical explanation to children’s misbehavior either by health institutional, professional guidance or by media (Erlandsson, Lundin, & Punzi, 2016; Ponnou & Gonon, 2017). Moreover, it is somewhat astonishing that both researchers and practitioners so easily seem to accept the biomedical model of ADHD and perceive pharmacological solutions as appropriate (Erlandsson & Punzi, 2016). Timimi (2005); Timimi & Leo (2009)) argues that the expansion of diagnosis as ADHD and the increase of prescriptions of stimulant medications is not an indication of a scientific progress rather a sign of how children in neo-liberal cultures are perceived. It has not been proven that medication as a treatment for children with ADHD is superior to a non-medical approach (Jensen et al., 2007). Follow-up studies (3 years or more after medication starts) do not show that long-term use of stimulants given to children with ADHD improve the condition when compared to non-medicated children with the same diagnosis.

Lakhan and Kirchgessner (2012) stress the need for long-term investigations and education regarding long-term health risks related to misuse of stimulants. According to the authors, such misuse may lead to serious physical and mental consequences like psychosis, myocardial infarction, cardiomyopathy and sudden death. In our view the increase of biomedical markers and solutions to children’s behavior problems rejects the impact of attachment and parenting and how early, object relations leave imprints on children’s brain functions and regulation of emotions. Furthermore, research has demonstrated that adjustment difficulties were more likely to occur among 1- to 3-year-olds who witnessed violence towards a family member in comparison to children who do not witness such violence (McDonald, Jouriles, Rosenfield, Briggs-Gowan, & Carter, 2007). Children who witness angry adult verbal conflict are also at risk for exhibiting adjustment difficulties, although to a lesser degree than when witnessing violence. The authors request longitudinal studies for the examination of family conflict and violence for a better understanding of how these dysfunctional characteristics of family life affect children. Altogether, family conflicts and violence witnessed by small children call for more thorough investigations of the reason behind children’s conduct problems.

In this study, our purpose was to investigate French digital communities that attract parents of children diagnosed with ADHD or who have behavioral problems that resemble ADHD. The aim was to study how parents affected by their children’s conduct present their daily hardships to other parents and professionals on Internet. Further, we aimed to apprehend the nature of the relationships found between parents’ online communities and health professionals, remaining aware of the active role that characterizes the contemporary links between patients and the medical field. The qualitative data (texts), collected from two Internet communities devoted to the parents of children diagnosed with ADHD, was analyzed using Grounded Theory. This method proposes that one core category can account for multiple discourses, whose main implications are integrated with one another (e.g., parents’ testimonials, health professionals’ advice, active members’ information, etc.). Furthermore, Grounded Theory makes it conceivable to highlight dimensions of social interaction, of which individuals may not be fully aware. Thereby, the analysis will lead us to reflect upon implications that digital communities themselves may not have considered in their discourse. Eventually, this may allow us to adopt a standpoint and suggest that parents begin to view their children’s restlessness and relational difficulties from a wider perspective.

## Materials and methods

### Data collection

The empirical data for this study include texts from two French Internet sites aimed at the parents of children with ADHD. Googling the keywords “ADHD” and “parent,” we identified the two Internet sites, which were fully dedicated to the questions parents may have about the disorder. More occasional references to ADHD found at general health or education sites were not included in the study material. The analysis, following the Grounded Theory method and influenced largely by Strauss and Corbin (1998), focused on the written content of these two digital communities. For a consistent and smooth analysis of the two sites, the content of each section and sub-section was copied into Word, with no major impact on the formatting differences of the output. For ethical reasons, all quotations used for the analysis were anonymized, and no information was provided that would distinguish one community from the other. The printed material allowed ND and MH to start the coding procedure, with further comparison and integration of the analysis through the fulfilment of the procedure described below. Table I presents examples of the sort of text that appeared in sections of the two sites, along with information offered to the users.

**Table I.** Examples of sections and type of information proposed to the users of the websites.

Section examples	Type of proposed information
Attention Deficit Hyperactivity Disorder (ADHD)	List of behaviors indicating a disorder, diagnosis approach, history of the disorder, associated disorders, examples of care, scientific and general, public information
ADHD at school	School adjustments, guidance for teachers, administrative recognition of the handicap, strategies for the management of concentration and relational difficulties in children
ADHD among adults	Consequences of ADHD on life situation, testimonials of adults diagnosed with ADHD, examples of care, information on books and scientific articles which aim at explaining ADHD to a lay audience
Current agenda	Links to social networks related to ADHD, television programs, newspaper articles, scientific events (e.g., conferences on the disorder) which promote the recognition of ADHD in children and adults
Practical information	Volunteers by region, resource institutions for the acknowledging of the handicap of an ADHD diagnosis

### Data analysis

The Grounded Theory method was used for carrying out a systematic analysis of data, aimed at generating a theory that reveals the main concern for individuals involved in the study (Glaser & Strauss, 1967). According to Glaser (1978) a core category provides the most consistency when doing this sort of analysis, accounting for the individuals’ main concern and how they deal with it. A three-step procedure was performed in accordance with recommendations formulated by Strauss and Corbin (Strauss & Corbin, 1998). As a first step the downloaded written material was subjected to open coding, which means a line-by-line coding for generating concepts considered to be close to the data. Open coding enabled us to clarify the concerns underlying digital messages sent by the members (e.g., “informing other parents,” “legitimating a medical approach,” “warning of inappropriate practices”). While carrying out open coding, the drafting of memos led to structuring a limited number of axes for the analysis. This axial coding assisted the progress in categorization of an underlying pattern of actions. Conditions and consequences of the sent messages were linked through memo-writing, recording a constant comparison of the concepts that were generated. Further comparisons of axes of analysis through more theoretical memos led to the refinement of a core category that in turn enabled a broader scope of the multiple concerns to emerge. A last step of selective coding consisted of rearranging the categories for integration and consistency of the analysis. The study’s findings are presented in light of the core category. A summary of the three-step procedure that was used is shown in Table II.

**Table II.** Stages of the analytical procedure following the approach of Strauss and Corbin (1998).

Stages of analysis	Description of the procedure
Open coding	Coding of the material line by line, aiming at clarifying the meaning of the message, its discursive effects, and its conditions of implementation
Axial coding	Structuring of generated concepts (i.e., open coding) around as few axes as possible through memo-writing for the identification of an underlying consistency in the individuals’ concerns and actions
Selective coding	Integrating identified axes of actions (i.e., axial coding) into an overarching category, which shapes and contributes to a broader scope of the study findings.

## Results

### Liberating parents from guilt

The emergence of a core category: “Liberating parents from guilt,” illustrates the underlying main concern behind the messages posted at the parents’ Internet sites. This main concern has to be continually agreed upon by parents, and by professionals who contribute to the digital communities as so-called experts on ADHD. The liberation of parents’ guilt starts by promoting a medical recognition of the child’s relational difficulties. Moreover, it highlights the diversity of advice, information, and occasional requirements addressed to other parents, primary and secondary school teachers, as well as any potential health professional, in need of information on ADHD. The main concern also accounts for current events, presented on the Internet (e.g., scientific congresses, parents’ local meetings). In doing so, the message from the communities is that parenting confronted with ADHD requires specific skills that single it out from traditional parenting. This kind of parenting is expected to fulfil what is required for a child diagnosed with ADHD, starting with the professionals who should be involved: medical, educational and administrative specialists. The advice given is that professionals often have to be persuaded of the legitimacy of the diagnosis. Thus, the special parenting skills are characterized as complicated and greatly shaped by the child’s underlying disorder. To liberate parents from guilt, the main goal of the communities is to declare the importance of medical attention and expertise as a way of formalizing and legitimizing the disorder. Promoting behavioral experts who can help parents to identify the disorder and suggest appropriate conduct in the child is another main concern. The communities recognize that relational difficulties commonly occur, stating that traditional parenting skills are ineffective with children who are diagnosed with ADHD. Parents are also encouraged to struggle against feelings of guilt and shame. In posted testimonies, those feelings are projected onto professionals who are said to lack special training in ADHD, and who challenge the medical approach to these children’s difficulties. An overview of the findings of the study is presented in Table III.

**Table III.** Study findings including the core category, supporting categories, and their properties (i.e., issues that members of the communities are expected to handle).

LIBERATING PARENTS FROM GUILT		
Seeking medical attention for the child	Promoting behavioral expertise	Accounting for parenting struggles
Medical recognition of ADHD is ignored Legitimating medicalization of the disorder	Programming relationships to the child Ensuring program effectiveness	Putting one’s trust in Internet communities Fitting into a story

### Seeking medical attention for the child

Parents’ digital communities present ADHD as a neurological disorder that requires a diagnosis and medical treatment. “Seeking medical attention for the child” is the first category that highlights the communities’ drive to recognize ADHD as a brain disorder, demanding special parenting practices. It means that parents can be freed from guilt by acknowledging the need to struggle for legitimization of the child’s difficulties in opposition to health professionals, who, it is felt, rarely fulfil the expectations of the parents.

#### Medical recognition of ADHD is ignored

According to the communities the diagnostic aspects of ADHD are lacking in France. It is argued that statistics indicate that ADHD is greatly under-diagnosed, leaving many children and their families without appropriate treatment or institutional assistance. The communities stress that health professionals are not sufficiently trained in the specific care that would benefit a child with ADHD. The basic idea propagated by the communities seems to be that there is a lack of recognition and management of ADHD in France, compared to other countries, where the families are shown more respect.

“*… following the example of our better endowed European neighbors, five parents founded this web site to help families, spare them a tortuous therapeutic journey, and foster a better school and social integration, in the hope that the establishment of an early diagnosis could improve the future of these children in every part of life*.”

Parents feel that they are discredited due to their child’s misbehavior at school, and left without support from professionals. Teachers, who perceive the child as restless, boisterous or even provocative towards adults, tend to judge the parent of that child. According to the parents, there is a shortage of institutions and practitioners known to be skilled in the management of a child with ADHD. The professionals ignore the suffering of families who must wait too long for a consultation, depriving the child of a chance for early improvement. Due to lacking recognition of the neurobiological origin of ADHD, some parents feel that they are blamed for their child’s disorder. The online communities assert that their child’s problem is not educational, nor does it depend on the school situation, but is a problem related to the development of the brain. This is a rather common statement on the web sites:

“*Attention Deficit Hyperactivity Disorder is neurobiological. The genetic factors associated with this disorder are often considered essential in explaining its onset. Therefore, this neurological disorder causes significant difficulties in self-control, forming ideas, carrying out activities and avoiding behaviors that are involuntary and difficult to control*.”

In terms of stigmatization, the communities compare ADHD with autism, another developmental disorder, and inform parents that they are in no sense responsible for their child’s condition or behavior. They should be wary of professionals who may apply educative, psychiatric or psychoanalytic approaches to their child’s difficulties. Research programs, led by European or American universities, are presented as evidence of the current, scientific consensus on ADHD as a brain condition. Video-filmed interventions by so-called experts on ADHD encourage parents to make up their own mind about the origin of the child’s disorder.

#### Legitimizing medicalization of the disorder

Parents on the websites complain about French health professionals’ skepticism regarding the legitimacy of the ADHD diagnosis, despite scientific consensus in several other countries. They suggest that misinformed media and lobbies, lacking in credibility, transmit this unhelpful opinion. Scientology, whose sect-like features are debated in France, is mentioned as one of the sources of misinformation, claiming that ADHD is an artificial, diagnostic trend, not medically justified. Therefore, the communities must warn parents about scientologists’ unfortunate labelling of methylphenidate, the privileged treatment for the disorder, as “kiddy coke.” The communities need continuously to oppose such prejudices against the children seen to have difficulties focusing:

“*Unfortunately, we still find quite a lot of* ‘*bad information’ on the subject with the feeling, for some, that the increase in diagnoses can be compared to an epidemic, a craze, a trend* [*…*] *However, information on the disorder and its acknowledgement improves every day, and let’s hope that will continue*.”

According to parents’ online communities, the so-called socio-educative perspective on how a family is affected by a child’s ADHD underestimates the seriousness of the condition. Parents are encouraged to identify the disorder and help the child to make progress through appropriate treatments, such as speech therapy, psychomotor- and psycho-emotional education. Examinations by experts are suggested to reveal commonly associated problems in diagnosed children, such as dysgraphia, dyspraxia, dyslexia and emotional difficulties (e.g., anxiety, depression, loss of self-esteem). Generally, health professionals would not advise them to undertake those complementary examinations. Therefore, parents must provide specialists with detailed observations, completed questionnaires, and their pleas for opinions from medical expertise to identify the seriousness of the child’s condition. Furthermore, they are encouraged not to give up their search for an ADHD diagnosis, even if a general practitioner or a neuropsychologist has not legitimized it. To recognize ADHD as a medical condition can lead to an optimal treatment for the child. Considering the stigmatization that he/she has to face, the achievement of this goal should not preclude any option for treatment:

“*We are not pro-drugs, but we are mindful that hurting children can benefit from the best diagnosis and the most accurate treatment, medicinal if needed*. […] *These are children in great pain, often victims of a lack of understanding, or even of rejection from society or school*.”

Testimonials at the communities’ websites emphasize the positive influence of drugs on children with ADHD. The drugs enable children to exhibit their capacities, help them to focus their attention and limit restlessness. It is stressed that working side by side with experts can help parents better to estimate what kind of care a child with ADHD needs. International parent networks can provide support for the acknowledgement of ADHD in France, whose progress is claimed to be slower than that of other countries (such as the USA, the UK, or Belgium). Parent networks in more progressive countries are suggested as examples for the French communities to follow.

### Promoting behavioral expertise

The second main category, “Promoting behavioral expertise”, highlights the goal of parents’ communities: the recognition of ADHD as a brain disorder that is out of parental control. The onset of ADHD in children is merely seen as disruptive for family bonding, to the point of making parents helpless in the face of their child’s misconduct. Educative programs adapted to the emotional consequences of the disorder, should imbue a hope to re-establish the parent/child relationship, and to enable parents to rediscover their child, as she or he was prior to the behavioral problems.

#### Programming relationships to the child

According to the Internet communities, adapted care will only be accessible if the parents manage to get an ADHD diagnosis for the child. Thereby, parents are invited to establish a dialogue with their physician based on a list of difficult behaviors, to convince the physician of the seriousness of the child’s difficulties. As many practitioners lack appropriate training, parents need to know about the complexity of ADHD, and ask for further examinations. It is claimed that parents themselves are ultimately the most knowledgeable persons to record the first observations on which the diagnostic and management specialists can base their analysis. The communities invite parents to contribute to the unravelling of the child’s problems by collecting all required information that professionals need: observations made by teachers, medical- and neuropsychological evaluations. Parents are told to believe that they are credible enough and sufficiently informed to play an active role in the establishment of an ADHD diagnosis in their child.

Experts on ADHD invite parents to formalize their relationship with the children by giving them purposeful goals for dealing with the attention deficit problem. Children should be taught that they can be rewarded for their efforts to be attentive, and that there will be no rewards if they don’t try to increase their attention span:

“*This behavioral approach aims at taking action on the child’s environment, in order to change unwanted behaviors* […] *this approach promotes positive reinforcement, pauses, or a period of reflection, along with the withdrawal of privileges through the use of tokens, and may use a combination of these different means*.”

Parents are also advised to set up goals, which are believed positively to strengthen the relationship that was altered by the child’s misconduct. Difficulties for parents to make long-term plans and to manage the child’s impulsiveness can be solved daily through programs available via the Internet forums. Considering the fact that parents of children with ADHD often feel frustrated, they are encouraged to focus their attention on accessible goals. Furthermore, parents are enlightened on the mechanisms of frustration for helping them to maintain progress in the parent/child relationship. For example, they can reward the child, and make the child aware of the advantage of training for improvements. Parents are also made aware of their own behavior, i.e., rejecting the child. They can take part in a methodological validation of their daily efforts to manage the child’s behavior in a more desirable way, despite difficulties encountered in the parent-child relationship.

#### Ensuring program effectiveness

Mobilization of parents beyond the family circle is justified by the impact of ADHD on children’s daily behavior. Parents’ digital communities request specialized training, widely provided to many professionals practicing with children: general practitioners, paediatricians, school doctors, speech therapists, psychomotor specialists, and teachers. This broad management prospect, outlined by the communities, reflects the multiple consequences of a child’s behavior problems associated with ADHD:

“*ADHD affects all areas of life: school life, social life, family life, self-esteem. The impact of the disorder can be alleviated with the implementation of adapted therapeutic and educative strategies as early as possible*.”

As a consequence of the communities’ information, teachers are told to be more aware of the prevalence of ADHD in the classroom and to help health professionals to identify children whose behavior is suspected to be a sign of ADHD. To facilitate classroom observations, teachers are exposed to video clips of behaviors they should pay attention to (such as absent mindedness, restlessness, and so forth). Overall, the message is that children’s misconduct is not a lack of willpower or provocation, but the consequences of a medical disorder for which neither parent nor child is responsible. Teachers are advised to contact the concerned families regularly and to adjust their teaching to the children’s attention deficit:

(For teachers) “*Teaching must be adapted to the children’s disorders: just as a teacher sits visually impaired children in the front row and ensures that they wear their glasses, he/she must place hyperactive children in a favorable environment and adopt an attitude of openness to their difficulties*.”

It is proposed that teachers should use short-term goals for children with behavior problems, and not expose them to frustration. In order to help children to concentrate on their schoolwork they shouldn’t be too secluded from their classmates. However, a separate space in the class-room for a child who is hyperactive or emotionally overwhelmed can be necessary. In order to increase the self-esteem of children with ADHD, teachers are invited openly to praise them in front of other pupils. This strategy is suggested to strengthen the children’s ability to be attentive and to limit problematic behaviors linked to their many frustrations:

(For teachers) “*Positive reinforcement is both important and useful because it enables you to praise or reward the small signs of progress and efforts of the children, and thereby improve their self-image. One should notice when children are doing well and find opportunities to admire them and be proud of them*. […] *In addition, this motivation helps in increasing the level of attention in these children during assignments*.”

Educational adjustments, requested by parents’ communities, are in line with recent requirements from public authorities regarding educational strategies and orientation. Integration of pupils through the recognition of a handicap, boosting them in a pedagogical relationship, is in accordance with the expectations of the families of children with ADHD.

### Accounting for parenting struggles

Left with inappropriate professionals’ responses, and consequences of ADHD on family relationships, parents are likely to blame themselves for their child’s conduct. By gathering in associations, parents realize the importance of collective actions in promoting social change. The digital communities enable parents to share their parenting experience by being open to those who are willing to listen and to give advice. Eventually, worried parents are able to find the support that health professionals didn’t give, having underestimated the seriousness of the situation. But sharing parenting experiences of those problems also implies taking responsibility for the social perception of the diagnosis. Liberating parents from guilt requires that parents contextualize their individual experience as part of a broader, collective aspiration. Hence, “Accounting for parenting struggles”, our third main category, highlights the communities’ push for public recognition of ADHD as a brain disorder, the impact of which goes beyond individual families.

#### Putting one’s trust in internet communities

Parents who, overall, lack trust in health professionals find in the Internet forums a place where nobody contests their views. Searching for medical recognition from one institution to another has often been a challenging experience for them. Digital communities offer a venue for worried parents to provide mutual assistance in the context of distrusting current professionals’ practices. Beyond social debates surrounding the medical reality of ADHD, the forums allow the parents to disclose their daily suffering to each other:

“*This association came from an online discussion forum, where parents of children diagnosed with ADHD, or looking for a diagnosis, tried to pool their educative skills and leave social isolation behind. Most of them experienced years of medical wandering before obtaining a diagnosis, followed by an equally long therapeutic wandering*.”

Parents feel authorized to use the communities’ Internet sites for posting testimonials, in which they reveal their feelings of loneliness in different upsetting situations. They share with other parents their experiences of professionals’ skepticism towards the child’s difficulties and their parenting practices. Some had to struggle against flawed medical prescriptions and side effects that didn’t seem to alarm the practitioners. There are examples of parents who spent several years with no help from health professionals or proposed treatment for their child’s behavioral problems:

“*One day, with tears in my eyes, I called back the health institution that diagnosed our child, asking them if they could do something… but no, they could do nothing. So, during his crises my son continued banging his head on the floor, saying he was stupid, gaining momentum from his bed and throwing himself against his bedroom door* […] *So I looked for a solution, and found a forum for parents of children with ADHD, and there I was given contact information for an association*.”

By showing that medical treatment of ADHD is progressing, parents’ online communities wish to bring hope to those who have not yet found a solution for their child. Methylphenidate (e.g., Ritaline) is said to help some children to become calmer and more focused. From being boisterous and rejected by other people including teachers, the child became better behaved, even beyond the behavior of the average pupil in the class. It is a priority to inform members of the communities of ways to find medical assistance and appropriate care for their child. No one in the digital communities disputed the need for medical treatment for a child diagnosed with ADHD.

#### Fitting into a story

The message sent by the forums is that parents are by far the most knowledgeable people about ADHD and its implications, making them the best advocates for their child. Having a child with ADHD means that you have responsibilities, even to other children with the same diagnosis. The communities suggest that each parent can contribute to broader social change, first instigated via the Internet by a few parents. Making public the scientific knowledge on how to take care of a child with ADHD, and promoting the relevant training of practitioners, is said to contribute to ongoing, positive change:

“*Our association’s objective is to strengthen the knowledge, screening, and care of the disorder through an educational action plan aimed at public authorities, the educational world, and the media*.”

The Internet communities’ ambition is to reduce the time that families have to wait for a follow-up, expert consultation of a child diagnosed with ADHD. Parents’ long struggle for their child’s welfare is presented in a historical and medical perspective—going back more than 150 years. It is stressed that ADHD, to this day, has been under-diagnosed, and that researchers now consider ADHD to be a genetic condition that exists from birth. However, the signs of it appear progressively during the developmental phase of the child. Parents are therefore encouraged to identify their child’s problematic behavior as soon as possible. Considering the obstacles to social acknowledgement, parents are also prompted to be respectful of collective actions led by others. They are reminded to measure and control their own personal expression on social media, ensuring the credibility of the messages published online.

“[The forum] *aims at helping, and any misuse or malice could lead to its closing. We therefore ask you to exercise respect and common courtesy in the exchanges and, if that is repeatedly not respected, we could, in a worst-case scenario, exclude you from this discussion space*.”

The individual story of parents’ experiences is expected to fit into a collective history for the recognition of ADHD by official authorities starting several years ago. Most changes in mentalities and practices are seen as the result of initiatives taken by parents in different parts of the world. Therefore, parents in the Internet communities are advised to participate in a move towards social progress that is in the best interest of all children diagnosed with ADHD.

## Discussion

As pointed out by Adrian (2014), the pathway to caring for parents who are concerned with their child’s diagnosis is as much an indicator of the beliefs of professionals they meet as an indicator of their own therapeutic needs. This also characterizes parents’ communities, which fully agree with the ADHD experts’ advice. The recommendations to establish an ADHD diagnosis drive parents to take a pro-active role in the management of their child. This advice is similar to the message in a best-selling, self-help book intended for the parents of children with ADHD. Barkley (2013) underscores the importance of the parent’s engagement and fighting for the child in all aspects of life:

“In areas where any reasonable and competent parent *wishes* to be involved in child rearing, parents of a child with ADHD *must* become involved—doubly involved. They must search out schools, teachers, professionals, and other community resources. They will find themselves having to supervise, monitor, teach, organize, plan, structure, reward, punish, guide, buffer, protect, and nurture their child far more than is demanded of a typical parent.” (Barkley, 2013, p. 5, original italics)

Right from the preface of his book, the author warns parents about the harmfulness of ADHD for the whole family relationship “if they do not engage in a specific kind of parenting” that is claimed to be suitable in managing a child with the disorder (Barkley, 2013, p. xi). With this mandate, the adult is invited to become “an executive parent” to his child and “the pivotal person who coordinates” caring activities. “Heroism” is required to fulfil this mission and parents are told never to let anyone take on their parental role. Our study shows that parents’ Internet communities may embrace these ideas.

Besides medical consideration, parents are usually advised to favour the autonomy of the child and enable her or him to try out the links between a choice and its consequence. Furthermore, they are advised to allow for emotions to be expressed without controlling the child’s emotional expression (Pajo & Stuart, 2012). Confronting misconduct in the relationship with the child (e.g., lack of attention to what the adult is saying, refusal to go to bed or to tidy up), can be solved through a dialogue with the child. This strategy is seen as preferable to an excessive, disciplinary control of the child’s conduct. The same problematic conduct, however, is presented to participants in parents’ Internet communities as requiring specific attention. They are advised to control the child through discipline and schedule activities in a directive way, which implies to adopt educative strategies opposite to those used for healthy children (Pajo & Stuart, 2012). The communities present an enormous amount of advice and guidance for ways that parents can foster and treat their atypical, abnormal child. As a consequence, the parent is placed in the position of an observer of conduct, rather than being a parent interacting with a child who needs to be recognized as a unique individual. Parents are neither invited to be spontaneous in their relationship with the child, nor to contextualize his or her impulsiveness. They are instead encouraged not to personalize the so-called manifestations of the neurological disorder (Barkley, 2013). The investigated communities’ endeavours to liberate parents from feelings of guilt may contribute to making them ignorant of family and marital contexts of their child’s conduct. The recognition of a biological aetiology is a major demand for the acknowledgement of ADHD as a neurodevelopmental, medical disorder. A narrative embracing the origin of ADHD as biological can give some relief to parents, who experience that they failed in parenting their child (Ghosh et al., 2016). An ADHD diagnosis may also help to restore the legitimacy of a parent who feels guilty for the child’s misconduct (Hansen & Hansen, 2006).

Examples of an alternative attitude to children’s misconduct problem can be found in many countries and cultures. An Iranian study, for instance, reveals that one out of two parents does not consider ADHD in their child as biological, and less than one out of ten sees their child’s misconduct as inevitably persisting in the future (Ghanizadeh, 2007). A study, performed in England, demonstrates that few fathers of children diagnosed with ADHD agree with either the need for medication or increased monitoring by parents, while their wives are mostly convinced that these strategies are useful (Singh, 2003). Being able to identify with the impulsive conduct of their sons, fathers do not consider these acts as pathological. Some parents do not overtly oppose this view, because they don’t want to fuel a conflict in a couple when there is a difference in opinion (Singh, 2003). According to the author, parenting associated with ADHD presented by parents’ communities should be understood and investigated as a form of social construction. Digital communities cannot guarantee that they effectively protect parents from feeling guilty (Muris et al., 2015; Muris & Meesters, 2012) merely by liberating parents from guilt feelings through an on-line discourse. Our study suggests that the numerous requirements for being an ultimate parent for a child with ADHD, instead, are likely to heighten feelings of guilt in those who don’t succeed to follow the guidelines. Furthermore, our results suggest that the both communities’ advice is paradoxical in suggesting that parents behave with their own child as if they were professionals. The guidelines promote a kind of education that favours a mirroring relationship with the “hyperactive” child, who is described as demanding attention, being restless, being rejected and poorly understood by others. Members of the communities are continually searching for new information in different forums, feeling rejected and poorly understood by professionals.

Research focusing on the implications of parent-child interactions for children’s well-being [e.g., (Lee et al., 2013; Schroeder & Kelley, 2009) is absent in the parents’ websites. Thus, parents are not told that relationships among members of a family may influence emotional regulation and impulse control in children. According to Harvey et al. (1997), this influence has been shown when parents share the same educative views. For example, when fathers feel more supported by their wives in the exercise of authority (Singh, 2003), or when children are less exposed to marital conflicts (Schroeder & Kelley, 2009). In the same way, parents would be unaware of the augmentation of early opposition to authority as a reaction to conflicts between couples and family discord (Harvey et al., 2011). In our study, digital communities tend to spread information which seems to exclude any reference to marital and family bonds. Ignoring the importance of family bonding and relationships is alarming since an increased number of parents who can’t find support from their own parents, relatives and friends are now looking for advice online (Plantin & Daneback, 2009).

Building trustful relationships with parents who are in a similar situation is a significant implication of the communities that should not be underestimated. Those who feel lonely and a lack of understanding are allowed to share their story in correspondence with other members of the communities. Faith in the community can be established through the unconditional terms of participation without moral judgement (Lawton et al., 2005). It seems as though the forums meet the needs of a dialogue between parents who share their experience of parenting a child with behavior problems. This need for a dialogue around parenting practices reflects a somewhat surprising observation regarding the literature on ADHD. A minority of studies, according to Singh (2003), involves fathers as participants, i.e., more than 90% of the studies refer to mothers under the label children’s “parents.” This might illustrate difficulties encountered by fathers to find their place in the ADHD scope of parenting styles. It broadly illustrates eventual difficulties in the couples’ dialogue around their child’s misconduct. An important recommendation for parents’ ADHD communities would be to include both mothers and fathers to take part in the web-based dialogue.

## Conclusions

The discourse of two digital communities of French parents for ADHD acknowledgement was analyzed by implementing Grounded Theory, a well-known qualitative approach. “Liberating parents from guilt” was found to be the underlying theme of the communities included in the study, manifested through the limited number of posts referring to research that does not embrace a medical discourse. The objectives of the communities are to remove feelings of guilt in suffering parents by emphasizing a presumed genetic origin of ADHD. It seems, however, that this theme is instead likely to increase the parents’ burden. In order to alleviate latent guilt feelings, parents are likely to feel the necessity to conduct themselves like professionals with their children (e.g., use positive reinforcement). Considering a more typical approach to parenting, however, one may wonder if such constant attention to the child’s behavior, instead becomes an obstacle for the children to communicate authentically with their parents. Noticeable is that most of what parents share with each other on the Internet sites is what they need in order to become competent parents, freed from guilt for their children’s misconduct. Another side of the coin is the perspective of the child, who is “stuck in the pathology paradigm,” and will continue “to be shipped off to so-called experts in abnormality, such as doctors and psychiatrists” (Nilsson Sjöberg, 2016, p. 11). As stated by Nilsson Sjöberg (2016), although an ADHD diagnosis may act as a relief for both child and parent it is nevertheless disempowering for both the individual and society.

Any influence stemming from research showing the importance of family dynamics and parenting practices on children’s well-being is eliminated from the two websites. From time to time, children are expected to behave according to their own childish interests (Winnicott, 1958) and should be allowed not always to behave as family and educators expect. Children should also have the basic right not continually to communicate with, and react to their surrounding environment (Winnicott, 1963). Furthermore, one may ask to what extent children diagnosed with ADHD are able not to behave like a disabled child, by adopting a role complementary to that of their managing and protective parents (Pajo & Stuart, 2012)? These questions are still not part of the ADHD framework as a neurodevelopmental disorder, which remains ignorant of the relational consequences of its basic assumptions (Erlandsson et al., 2016).

Since the analysis is based on rather limited data, it cannot be claimed to be representative of parents’ digital communities in other countries. Hence, to challenge the framework that emerged in this study, future investigations need to be undertaken. To further investigate the relevance of our categories, a theoretical sampling procedure should follow (Draucker, 2007; Glaser, 1978). This can be done by including a broader diversity of data from parents’ communities for the recognition of ADHD in other parts of the world (see Smith, 2018). The medicalization of conduct problems occurring among children and adolescents has gone much too far and we need to choose treatment alternatives that can be modelled to the individual child. A clinical, non-diagnostic approach for enabling children and families to work with challenging behaviors is the Relational Awareness (RAP) developed and practiced by Timimi (2017). Instead of focusing on dysfunctions, and controlling behaviors and symptoms working with RAP means to approach children and their families as relational and emotional human beings. This treatment approach is a most relevant alternative to the mainstream biomedical solution that portray children as dysfunctional, and therefore in need of stimulants.
